# (*E*)-4-{2-[(4-Chloro­phen­yl)imino­meth­yl]phen­oxy}phthalonitrile

**DOI:** 10.1107/S1600536809011933

**Published:** 2009-04-10

**Authors:** Marife Tüfekçi, Gökhan Alpaslan, Ferda Erşahin, Erbil Ağar, Ahmet Erdönmez

**Affiliations:** a Department of Physics, Faculty of Arts & Science, Ondokuz Mayıs University, TR-55139 Kurupelit-Samsun, Turkey; b Department of Chemistry, Faculty of Arts & Science, Ondokuz Mayıs University, 55139 Samsun, Turkey

## Abstract

In the title compound, C_21_H_12_ClN_3_O, the phenoxy ring makes dihedral angles of 51.42 (5) and 65.01 (6)°, respectively, with the chlorophenyl and phthalonitrile rings. In the crystal structure, the mol­ecules are inter­linked through weak C—H⋯N and C—H⋯π contacts, and π–π stacking inter­actions *via* crystallographic inversion centres form a three-dimensional network. The distance between the centroids of the phthalonitrile rings is 3.9104 (11)Å, with a slippage between the rings of 1.626 Å and a perpendicular distance between the rings of 3.556 Å.

## Related literature

For the structure of dicyano­benzene, see: Janczak & Kubiak (1995 ▶). For the structure of 4-(2-formyl­phen­oxy)phthalonitrile and historical background to phthalocyanines and subphthalocyanines, see: Kartal *et al.* (2006 ▶).
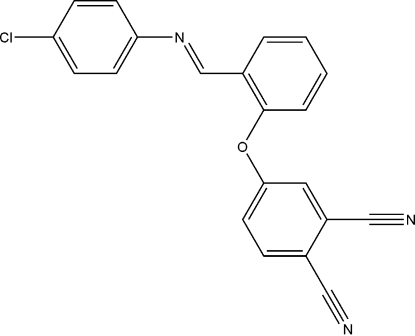


## Experimental

### 

#### Crystal data

C_21_H_12_ClN_3_O
*M* *_r_* = 357.79
Triclinic, 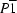

*a* = 8.8342 (9) Å
*b* = 10.2301 (8) Å
*c* = 11.2401 (9) Å
α = 76.473 (6)°
β = 84.912 (7)°
γ = 64.419 (6)°
*V* = 890.74 (13) Å^3^
*Z* = 2
Mo *K*α radiation
μ = 0.23 mm^−1^
*T* = 296 K
0.78 × 0.66 × 0.51 mm

#### Data collection

Stoe IPDS-II diffractometer
Absorption correction: integration (*X-RED32*; Stoe & Cie, 2002 ▶) *T*_min_ = 0.870, *T*_max_ = 0.904
8944 measured reflections
3503 independent reflections
2825 reflections with *I* > 2σ(*I*)
*R*_int_ = 0.044

#### Refinement

*R*[*F* ^2^ > 2σ(*F* ^2^)] = 0.045
*wR*(*F* ^2^) = 0.130
*S* = 1.04
3503 reflections
239 parameters
H atoms treated by a mixture of independent and constrained refinement
Δρ_max_ = 0.17 e Å^−3^
Δρ_min_ = −0.40 e Å^−3^

### 

Data collection: *X-AREA* (Stoe & Cie, 2002 ▶); cell refinement: *X-AREA*; data reduction: *X-RED32* (Stoe & Cie, 2002 ▶); program(s) used to solve structure: *SHELXS97* (Sheldrick, 2008 ▶); program(s) used to refine structure: *SHELXL97* (Sheldrick, 2008 ▶); molecular graphics: *ORTEP-3 for Windows* (Farrugia, 1997 ▶) and *PLATON* (Spek, 2009 ▶); software used to prepare material for publication: *WinGX* (Farrugia, 1999 ▶).

## Supplementary Material

Crystal structure: contains datablocks I, global. DOI: 10.1107/S1600536809011933/si2163sup1.cif

Structure factors: contains datablocks I. DOI: 10.1107/S1600536809011933/si2163Isup2.hkl

Additional supplementary materials:  crystallographic information; 3D view; checkCIF report

## Figures and Tables

**Table 1:** Hydrogen-bond geometry (Å, °)

*D*—H⋯*A*	*D*—H	H⋯*A*	*D*⋯*A*	*D*—H⋯*A*
C6—H6⋯N1^i^	0.93	2.60	3.521 (3)	172
C2—H2⋯*Cg*3^ii^	0.93	2.89	3.7044 (18)	148

Symmetry codes: (i) 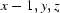; (ii) 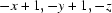. *Cg*3 is the centroid of the chloro­phenyl ring C16–C21.

**Fig. 1.**
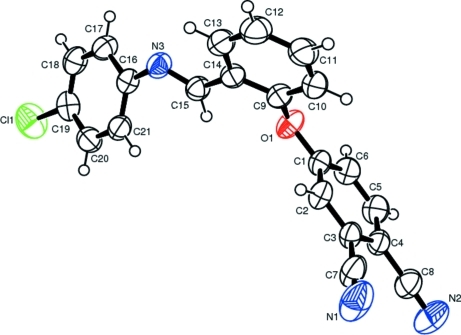
The molecular structure of (I), showing the atom-numbering scheme. Displacement ellipsoids are drawn at the 50% probability level and H atoms are shown as small spheres of arbitrary radii.

**Fig. 2.**
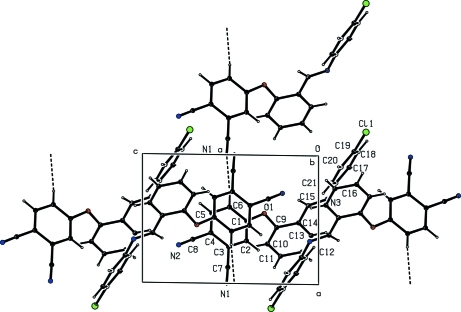
Part of the crystal packing of (I) viewed down the b axis. The partial stacking between the phthalonitrile rings in the centre of the unit cell is shown, and the weak C6—H6···N1i hydrogen bonding contacts (Table 1) are indicated by dashed lines.

## supplementary crystallographic information

### Comment

Substituted phthalonitriles are generally used for preparing symmetrically and unsymmetrically peripherally and non- peripherally substituted phthalocyanines and subphthalocyanines. In addition to their extensive use as dyes and pigments, phthalocyanines have found widespread application in catalysis, in optical recording, as photoconductive materials, in photodynamic therapy and as chemical sensors (Kartal *et al.* 2006 with literature cited therein).

The geometry of the phthalonitrile group in the title compound (Fig. 1), agrees with that of previously reported structures (Janczak & Kubiak, 1995; Kartal *et al.*, 2006). The values of the two C—O bond lengths are consistent with those found in a similar compound (Kartal *et al.*, 2006). Rings A (atoms C16 - C21) and B(C9 - C14) have a dihedral angle of 51.42 (5)°. The molecule is not planar, the dihedral angle between the phthalonitrile moiety and the ring B(C19 - C14) being 65.01 (6)°.

The unit cell of the structure of (I) (Fig. 2) shows an intermolecular π–π contact between the two symmetry related phthalonitrile rings of neighbouring molecules. The centre of gravity *Cg*1 of the ring C1–C6 has a perpendicular distance to *Cg*1^i^ of 3.556Å [symmetry code (i): 1 - *x*, 1 - *y*, 1 - *z*]. The distance between the ring centroids is 3.9104 (11) Å, with a slippage between the rings of 1.626 Å. Furthermore, the molecules are linked through weak intermolecular C—H···N contacts to form chains along the *a* axis (Fig. 2). These chains are connected *via* inversion related π–π interactions given above, and together with C—H···π contacts (Table 1) a three-dimensional network is formed. *Cg*3 is the centroid of the chlorophenyl ring C16 - C21.

### Experimental

To a solution of Salicylaldehyde (0.5 g, 4.09 mmol) in DMF was added potasium carbonato (1.12 g, 8.18 mmol). The mixture was stirred for 30 min under N2. 4-Nitrophtalonitrile (0.71 g, 4.09 mmol) solution in DMF was added. The mixture was stirred for 48 h at 323 K under N2 and poured into ice-water (150 g). The product 2-(3,4-Dicyanophenoxy) benzaldehyde was filtered off and washed with water. The title compound (I) was prepared by reflux a mixture of a solution containing 2-(3,4-Dicyanophenoxy) benzaldehyde (0.5 g 2.016 mmol) in 20 ml e thanol and a solution containing 2-Chloroaniline (0.257 g 2.016 mmol) in 20 ml e thanol. The reaction mixture was stirred for 1 h under reflux. The crystals of the title compound were obtained from ethylalcohol by slow evaporation (yield % 51; m.p.409–411 K).

### Refinement

The H atom bonded to C15 was refined freely. All other H atoms were placed in calculated positions and constrained to ride on their parent atoms, with C—H = 0.93–0.97 Å and *U*_iso_(H) = 1.2 *U*_eq_(C) or 1.5*U*_eq_(methyl C).

### Crystal data

**Table:**

C_21_H_12_ClN_3_O	*Z* = 2
*M**_r_* = 357.79	*F*(000) = 368
Triclinic, *P*1	*D*_x_ = 1.334 Mg m^−^^3^
Hall symbol: -P 1	Mo *K*α radiation, λ = 0.71073 Å
*a* = 8.8342 (9) Å	Cell parameters from 6275 reflections
*b* = 10.2301 (8) Å	θ = 1.9–28.1°
*c* = 11.2401 (9) Å	µ = 0.23 mm^−^^1^
α = 76.473 (6)°	*T* = 296 K
β = 84.912 (7)°	Block, colorless
γ = 64.419 (6)°	0.78 × 0.66 × 0.51 mm
*V* = 890.74 (13) Å^3^	

### Data collection

**Table:**

Stoe IPDS-II diffractometer	3503 independent reflections
Radiation source: fine-focus sealed tube	2825 reflections with *I* > 2σ(*I*)
graphite	*R*_int_ = 0.044
Detector resolution: 6.67 pixels mm^-1^	θ_max_ = 26.0°, θ_min_ = 1.9°
ω scans	*h* = −10→10
Absorption correction: integration (*X-RED32*; Stoe & Cie, 2002)	*k* = −12→12
*T*_min_ = 0.870, *T*_max_ = 0.904	*l* = −13→13
8944 measured reflections	

### Refinement

**Table:**

Refinement on *F*^2^	Primary atom site location: structure-invariant direct methods
Least-squares matrix: full	Secondary atom site location: difference Fourier map
*R*[*F*^2^ > 2σ(*F*^2^)] = 0.045	Hydrogen site location: inferred from neighbouring sites
*wR*(*F*^2^) = 0.130	H atoms treated by a mixture of independent and constrained refinement
*S* = 1.04	*w* = 1/[σ^2^(*F*_o_^2^) + (0.0664*P*)^2^ + 0.1064*P*] where *P* = (*F*_o_^2^ + 2*F*_c_^2^)/3
3503 reflections	(Δ/σ)_max_ < 0.001
239 parameters	Δρ_max_ = 0.17 e Å^−^^3^
0 restraints	Δρ_min_ = −0.40 e Å^−^^3^

### Special details

**Table:**

Geometry. All e.s.d.'s (except the e.s.d. in the dihedral angle between two l.s. planes) are estimated using the full covariance matrix. The cell e.s.d.'s are taken into account individually in the estimation of e.s.d.'s in distances, angles and torsion angles; correlations between e.s.d.'s in cell parameters are only used when they are defined by crystal symmetry. An approximate (isotropic) treatment of cell e.s.d.'s is used for estimating e.s.d.'s involving l.s. planes.
Refinement. Refinement of *F*^2^ against ALL reflections. The weighted *R*-factor *wR* and goodness of fit *S* are based on *F*^2^, conventional *R*-factors *R* are based on *F*, with *F* set to zero for negative *F*^2^. The threshold expression of *F*^2^ > σ(*F*^2^) is used only for calculating *R*-factors(gt) *etc*. and is not relevant to the choice of reflections for refinement. *R*-factors based on *F*^2^ are statistically about twice as large as those based on *F*, and *R*- factors based on ALL data will be even larger.

### Fractional atomic coordinates and isotropic or equivalent isotropic displacement parameters (Å^2^)

**Table:**

	*x*	*y*	*z*	*U*_iso_*/*U*_eq_	
C1	0.51280 (17)	0.26143 (17)	0.40778 (14)	0.0464 (3)	
C2	0.66702 (18)	0.26554 (19)	0.40930 (14)	0.0505 (4)	
H2	0.7379	0.2495	0.3427	0.061*	
C3	0.71421 (18)	0.29367 (18)	0.51070 (13)	0.0475 (4)	
C4	0.6080 (2)	0.31856 (18)	0.61105 (14)	0.0497 (4)	
C5	0.4538 (2)	0.3153 (2)	0.60666 (16)	0.0602 (4)	
H5	0.3817	0.3324	0.6725	0.072*	
C6	0.4063 (2)	0.2869 (2)	0.50597 (16)	0.0560 (4)	
H6	0.3024	0.2847	0.5039	0.067*	
C7	0.8749 (2)	0.2967 (2)	0.51519 (16)	0.0661 (5)	
C8	0.6607 (3)	0.3483 (2)	0.71432 (17)	0.0696 (5)	
C9	0.55995 (19)	0.14409 (18)	0.23769 (15)	0.0497 (4)	
C10	0.6889 (2)	0.0109 (2)	0.28960 (17)	0.0606 (4)	
H10	0.7120	−0.0135	0.3731	0.073*	
C11	0.7829 (2)	−0.0855 (2)	0.21644 (19)	0.0678 (5)	
H11	0.8710	−0.1751	0.2505	0.081*	
C12	0.7476 (2)	−0.0504 (2)	0.09301 (19)	0.0678 (5)	
H12	0.8110	−0.1166	0.0442	0.081*	
C13	0.6185 (2)	0.0823 (2)	0.04203 (16)	0.0583 (4)	
H13	0.5952	0.1051	−0.0413	0.070*	
C14	0.52227 (18)	0.18339 (18)	0.11312 (15)	0.0490 (4)	
C15	0.38617 (18)	0.32668 (19)	0.05899 (15)	0.0486 (4)	
C16	0.20883 (18)	0.49755 (18)	−0.09784 (13)	0.0474 (4)	
C17	0.0858 (2)	0.5086 (2)	−0.17321 (15)	0.0550 (4)	
H17	0.0871	0.4229	−0.1892	0.066*	
C18	−0.0379 (2)	0.6449 (2)	−0.22445 (15)	0.0590 (4)	
H18	−0.1210	0.6519	−0.2742	0.071*	
C19	−0.0371 (2)	0.7704 (2)	−0.20116 (16)	0.0576 (4)	
C20	0.0837 (2)	0.7630 (2)	−0.12803 (16)	0.0584 (4)	
H20	0.0827	0.8492	−0.1134	0.070*	
C21	0.2069 (2)	0.62566 (19)	−0.07630 (14)	0.0529 (4)	
H21	0.2895	0.6194	−0.0265	0.063*	
N1	1.0006 (2)	0.2993 (3)	0.52178 (18)	0.1025 (8)	
N2	0.7057 (3)	0.3728 (3)	0.79419 (19)	0.1125 (8)	
N3	0.33454 (16)	0.35400 (16)	−0.04982 (12)	0.0533 (3)	
O1	0.45285 (13)	0.24132 (14)	0.30878 (11)	0.0594 (3)	
Cl1	−0.19019 (7)	0.94362 (6)	−0.26930 (6)	0.0948 (2)	
H15	0.346 (2)	0.3985 (19)	0.1112 (15)	0.048 (4)*	

### Atomic displacement parameters (Å^2^)

**Table:**

	*U*^11^	*U*^22^	*U*^33^	*U*^12^	*U*^13^	*U*^23^
C1	0.0430 (7)	0.0486 (8)	0.0424 (8)	−0.0151 (6)	−0.0058 (6)	−0.0067 (6)
C2	0.0435 (7)	0.0656 (10)	0.0384 (8)	−0.0198 (7)	−0.0002 (6)	−0.0099 (7)
C3	0.0451 (7)	0.0522 (9)	0.0407 (8)	−0.0187 (6)	−0.0062 (6)	−0.0033 (6)
C4	0.0576 (8)	0.0479 (8)	0.0400 (8)	−0.0190 (7)	−0.0022 (6)	−0.0088 (6)
C5	0.0592 (9)	0.0692 (11)	0.0505 (10)	−0.0254 (8)	0.0144 (7)	−0.0188 (8)
C6	0.0461 (8)	0.0651 (10)	0.0565 (10)	−0.0247 (7)	0.0036 (7)	−0.0116 (8)
C7	0.0583 (10)	0.0966 (15)	0.0460 (9)	−0.0378 (10)	−0.0084 (7)	−0.0072 (9)
C8	0.0874 (13)	0.0719 (12)	0.0501 (10)	−0.0316 (10)	0.0007 (9)	−0.0187 (9)
C9	0.0487 (8)	0.0539 (9)	0.0496 (9)	−0.0237 (7)	−0.0056 (6)	−0.0109 (7)
C10	0.0683 (10)	0.0553 (10)	0.0557 (10)	−0.0246 (8)	−0.0160 (8)	−0.0046 (8)
C11	0.0736 (11)	0.0454 (9)	0.0765 (13)	−0.0160 (8)	−0.0191 (9)	−0.0097 (9)
C12	0.0722 (11)	0.0550 (10)	0.0739 (12)	−0.0190 (9)	−0.0057 (9)	−0.0229 (9)
C13	0.0639 (10)	0.0570 (10)	0.0555 (10)	−0.0246 (8)	−0.0062 (7)	−0.0143 (8)
C14	0.0488 (8)	0.0511 (8)	0.0500 (9)	−0.0244 (7)	−0.0059 (6)	−0.0077 (7)
C15	0.0470 (7)	0.0531 (9)	0.0474 (8)	−0.0224 (7)	−0.0034 (6)	−0.0102 (7)
C16	0.0469 (7)	0.0539 (9)	0.0399 (8)	−0.0203 (7)	0.0010 (6)	−0.0099 (6)
C17	0.0555 (9)	0.0580 (10)	0.0520 (9)	−0.0216 (7)	−0.0064 (7)	−0.0152 (7)
C18	0.0483 (8)	0.0714 (11)	0.0515 (9)	−0.0208 (8)	−0.0065 (7)	−0.0094 (8)
C19	0.0483 (8)	0.0565 (10)	0.0532 (10)	−0.0138 (7)	0.0053 (7)	−0.0035 (7)
C20	0.0651 (10)	0.0535 (10)	0.0574 (10)	−0.0268 (8)	0.0075 (8)	−0.0131 (8)
C21	0.0557 (8)	0.0621 (10)	0.0438 (8)	−0.0277 (7)	−0.0014 (6)	−0.0109 (7)
N1	0.0731 (11)	0.175 (2)	0.0727 (12)	−0.0723 (14)	−0.0127 (9)	−0.0072 (13)
N2	0.160 (2)	0.130 (2)	0.0671 (13)	−0.0674 (17)	−0.0122 (13)	−0.0413 (13)
N3	0.0529 (7)	0.0549 (8)	0.0489 (7)	−0.0187 (6)	−0.0087 (6)	−0.0104 (6)
O1	0.0454 (6)	0.0776 (8)	0.0522 (6)	−0.0184 (5)	−0.0088 (5)	−0.0207 (6)
Cl1	0.0776 (4)	0.0632 (3)	0.1105 (5)	−0.0075 (3)	−0.0174 (3)	0.0041 (3)

### Geometric parameters (Å, °)

**Table:**

C1—O1	1.3663 (19)	C11—H11	0.9300
C1—C6	1.382 (2)	C12—C13	1.374 (2)
C1—C2	1.383 (2)	C12—H12	0.9300
C2—C3	1.377 (2)	C13—C14	1.392 (2)
C2—H2	0.9300	C13—H13	0.9300
C3—C4	1.399 (2)	C14—C15	1.468 (2)
C3—C7	1.439 (2)	C15—N3	1.269 (2)
C4—C5	1.383 (2)	C15—H15	0.975 (17)
C4—C8	1.428 (2)	C16—C21	1.380 (2)
C5—C6	1.373 (2)	C16—C17	1.387 (2)
C5—H5	0.9300	C16—N3	1.417 (2)
C6—H6	0.9300	C17—C18	1.375 (2)
C7—N1	1.132 (2)	C17—H17	0.9300
C8—N2	1.132 (3)	C18—C19	1.373 (3)
C9—C10	1.379 (2)	C18—H18	0.9300
C9—C14	1.392 (2)	C19—C20	1.370 (3)
C9—O1	1.3958 (19)	C19—Cl1	1.7420 (17)
C10—C11	1.375 (3)	C20—C21	1.381 (2)
C10—H10	0.9300	C20—H20	0.9300
C11—C12	1.378 (3)	C21—H21	0.9300
			
O1—C1—C6	116.38 (14)	C11—C12—H12	120.0
O1—C1—C2	122.92 (14)	C12—C13—C14	121.04 (17)
C6—C1—C2	120.58 (15)	C12—C13—H13	119.5
C3—C2—C1	119.13 (14)	C14—C13—H13	119.5
C3—C2—H2	120.4	C9—C14—C13	117.61 (14)
C1—C2—H2	120.4	C9—C14—C15	120.99 (15)
C2—C3—C4	120.84 (14)	C13—C14—C15	121.40 (15)
C2—C3—C7	120.21 (14)	N3—C15—C14	121.16 (15)
C4—C3—C7	118.95 (15)	N3—C15—H15	124.2 (10)
C5—C4—C3	118.85 (15)	C14—C15—H15	114.5 (10)
C5—C4—C8	121.86 (15)	C21—C16—C17	119.08 (15)
C3—C4—C8	119.28 (15)	C21—C16—N3	123.10 (14)
C6—C5—C4	120.58 (14)	C17—C16—N3	117.79 (15)
C6—C5—H5	119.7	C18—C17—C16	120.62 (17)
C4—C5—H5	119.7	C18—C17—H17	119.7
C5—C6—C1	120.02 (15)	C16—C17—H17	119.7
C5—C6—H6	120.0	C19—C18—C17	119.06 (16)
C1—C6—H6	120.0	C19—C18—H18	120.5
N1—C7—C3	178.2 (2)	C17—C18—H18	120.5
N2—C8—C4	178.1 (2)	C20—C19—C18	121.59 (16)
C10—C9—C14	121.69 (15)	C20—C19—Cl1	119.19 (15)
C10—C9—O1	121.60 (15)	C18—C19—Cl1	119.20 (14)
C14—C9—O1	116.54 (13)	C19—C20—C21	119.01 (17)
C11—C10—C9	119.18 (16)	C19—C20—H20	120.5
C11—C10—H10	120.4	C21—C20—H20	120.5
C9—C10—H10	120.4	C16—C21—C20	120.64 (15)
C10—C11—C12	120.48 (17)	C16—C21—H21	119.7
C10—C11—H11	119.8	C20—C21—H21	119.7
C12—C11—H11	119.8	C15—N3—C16	117.86 (14)
C13—C12—C11	119.99 (17)	C1—O1—C9	120.84 (11)
C13—C12—H12	120.0		
			
O1—C1—C2—C3	176.80 (14)	C12—C13—C14—C9	−0.9 (3)
C6—C1—C2—C3	0.7 (2)	C12—C13—C14—C15	178.86 (17)
C1—C2—C3—C4	−0.3 (2)	C9—C14—C15—N3	−169.24 (16)
C1—C2—C3—C7	179.22 (16)	C13—C14—C15—N3	11.0 (2)
C2—C3—C4—C5	−0.3 (2)	C21—C16—C17—C18	−1.0 (2)
C7—C3—C4—C5	−179.85 (16)	N3—C16—C17—C18	−178.84 (15)
C2—C3—C4—C8	−179.60 (16)	C16—C17—C18—C19	0.8 (3)
C7—C3—C4—C8	0.9 (3)	C17—C18—C19—C20	−0.2 (3)
C3—C4—C5—C6	0.5 (3)	C17—C18—C19—Cl1	178.23 (13)
C8—C4—C5—C6	179.76 (17)	C18—C19—C20—C21	−0.2 (3)
C4—C5—C6—C1	−0.1 (3)	Cl1—C19—C20—C21	−178.63 (12)
O1—C1—C6—C5	−176.87 (15)	C17—C16—C21—C20	0.6 (2)
C2—C1—C6—C5	−0.6 (3)	N3—C16—C21—C20	178.31 (14)
C14—C9—C10—C11	0.1 (3)	C19—C20—C21—C16	0.0 (2)
O1—C9—C10—C11	175.30 (17)	C14—C15—N3—C16	−176.75 (14)
C9—C10—C11—C12	−0.8 (3)	C21—C16—N3—C15	39.8 (2)
C10—C11—C12—C13	0.6 (3)	C17—C16—N3—C15	−142.39 (16)
C11—C12—C13—C14	0.3 (3)	C6—C1—O1—C9	−145.81 (16)
C10—C9—C14—C13	0.7 (3)	C2—C1—O1—C9	38.0 (2)
O1—C9—C14—C13	−174.69 (14)	C10—C9—O1—C1	38.3 (2)
C10—C9—C14—C15	−179.06 (15)	C14—C9—O1—C1	−146.30 (15)
O1—C9—C14—C15	5.6 (2)		

### Hydrogen-bond geometry (Å, °)

**Table:**

*D*—H···*A*	*D*—H	H···*A*	*D*···*A*	*D*—H···*A*
C6—H6···N1^i^	0.93	2.60	3.521 (3)	172
C2—H2···Cg3^ii^	0.93	2.89	3.7044 (18)	148

Symmetry codes: (i) *x*−1, *y*, *z*; (ii) −*x*+1, −*y*+1, −*z*.
